# Aspects of young employees' mental health: a narrative review

**DOI:** 10.13075/ijomeh.1896.02787

**Published:** 2026

**Authors:** Aleksandra Marczak, Julia Piechota, Kaja Staszewska, Marta Wiszniewska

**Affiliations:** 1 University of Lodz, Institute of Psychology, Łódź, Poland; 2 Nofer Institute of Occupational Medicine, Unit of Health and Work Psychology, Łódź, Poland; 3 Nofer Institute of Occupational Medicine, Clinic of Occupational Diseases and Environmental Health, Łódź, Poland

**Keywords:** workplace, employment, young adults, narrative review, young employees, mental health

## Abstract

The mental health of employees has attracted an increasing degree of scientific attention. However, the majority of studies address the working population as a whole, with limited focus on young employees as a distinct group. The present narrative review synthesizes literature that was published in 2003–2025, which was identified through major online databases. This review focuses on young workers, most commonly defined in the literature as individuals aged 18–35 years who have entered the workforce within the past years. The aim of this review is to present the current state of knowledge with respect to the mental health of young workers, with particular attention to psychosocial risk factors contributing to this issue. The focus is on the differences between generations, the state of young employees’ mental health, psychosocial work factors connected to it and identifies 6 key psychosocial risk factors associated with poorer mental health outcomes: poor psychosocial job quality, exposure to sexual harassment, emotional workload and work pressure, subjective job insecurity, effort–reward imbalance with low social support, and precarious employment. In the analyzed studies, young workers appear to be a group at risk of mental disorders, with work-related stressors playing a significant role in their occurrence. The review also compiles practical organizational solutions aimed at improving job quality, stability, support and minimizing psychosocial risks.

## Highlights

- Young workers’ mental health affects productivity, absenteeism and labor market.
- Comprehensive organisational strategies can improve young employees’ mental health.
- Workplace risk factors are linked to the mental well-being of young workers.

## INTRODUCTION

The World Health Organization defines mental health as a state of well-being in which the individual realizes his or her own abilities, can cope with the normal stresses of life, can work productively and fruitfully, and is able to make a contribution to his or her community [1]. Over the years, the interest in mental health among people from different age groups, and in different contexts has increased. One of the groups that researchers often focus on when it comes to mental health is young adults. It seems understandable, according to the fact that 62.5% of onsets of any mental disorders appear before the age of 25 years [2]. Additionally, 1 study showed that only 51% of Generation Z (people born between 1997 and 2012) and 56% of Millennials (born between 1981 and 1996) assess their mental health as good or extremely good [3,4].

The state of mental health of this cohort can be viewed from many perspectives, one of them being work. According to WHO, 15% of working-age people have to deal with one of the mental disorders at any point in time [5]. Mental health problems may be viewed from the perspective of the whole working population, but an insight into particular age groups seems more appropriate. There are differences between younger and older generations, when it comes to the aspect of work. In various academic articles, different age groups are referred to as young workers, but most commonly this term is used for individuals who fall within the age range of 18–35 years and who have entered the workforce within the past several years [6–9]. Young employees face an increasingly more complex and difficult labor market situation, very different from previous generations. Their career trajectories may be described as unstable and changeable. A significant number of young people tend to go back and forth between employment, education and periods of unemployment [6]. Some young adults go through a series of temporary jobs with no prospect of promotion or long-term employment [10]. These kinds of jobs do not give them a sense of satisfaction. Such difficult conditions have the potential to threaten workers’ basic psychological needs, such as the need for security, autonomy, stability and control [6].

Taking those findings into consideration, it seems more than important to explore the topic of young employees’ mental health, as the transition into the labor market represents a critical life stage in which developmental, occupational, and social challenges accumulate. Mental health problems emerging at this stage may not only reduce individual well-being but also have long-term consequences for career trajectories, employment stability, and overall labor market functioning. Against this background, the aim of this article is to synthesize current knowledge on young employees’ mental health in order to elucidate the scope and complexity of the problem and to identify practical, evidence-based preventive and intervention strategies relevant to employers.

## METHODS

The literature review was conducted using online bibliographic databases such as Google Scholar, PubMed, Research Gate and EBSCO. The primary criterion for the selection of publications was the topic of the mental health of young employees’ (aged 18–35 years) and the factors connected to it. The timeframe of the work described in the used articles was 2003–2025. In addition, online sources were searched using the following keywords in different configurations: young employees’ mental health, young workers, young employees, young adults’ mental health, work stress in young adults, effects of mental health problems of young employees, psychosocial factors. Applying inclusion criteria related to the age range of young adults, employment status, and publication recency resulted in the inclusion of 41 articles concerning young workers, which were then classified according to their primary focus. These sources were categorized as follows: generational differences (N = 4), state of mental health (N = 4), psychosocial risks (N = 9), mobbing (N = 3), effects of mental health problems (N = 7), and preventive measures (N = 17). This categorization clearly indicates the thematic focus of the included studies.

The article is a narrative review that provides an overview of various studies on the mental health of young employees. Due to the niche nature of the issue, it incorporates studies employing various methodological approaches and conducted in different contexts. This approach enabled the integration of existing knowledge, the identification of key themes and research gaps, and the indication of areas requiring further analysis. It provides a reference point for future empirical research and more advanced systematic reviews.

## RESULTS

The mental health of young employees has many aspects. Researchers need to take a multifaceted approach to thoroughly understand the mechanisms concerning the problems of this cohort. From a scientific point of view, it is important not only to focus on the mental health of young employees itself, but also to learn about the factors that may be associated with their problems in this area. Possible measures that employers can implement to support the mental health of young employees are suggested in various publications that are now available.

### Generational differences in workplace

It is without doubt that generations differ when it comes to many aspects of life, one of them being psychological traits – and it shows in workplaces.

Research reveals a clear generational trend toward increased career mobility, with Millennials exhibiting the highest rates of job and organizational changes. Compared to previous generations, they are significantly more likely to switch roles, indicating a shift in workplace norms and expectations. This pattern is attributed to technological advancements, globalization, and economic fluctuations that demand greater adaptability from workers. As a result, modern careers are increasingly defined by flexibility and frequent transitions [11]. Millennials tend to show greater confidence and narcissism, along with increased levels of anxiety and depression. They care less about gaining approval from others [12]. What they care about mostly is flexibility, mobility, success and creativity. They do not tolerate monotony [13].

Generation Z seems to be even more demanding when it comes to their expectations in the workplace. It is the most connected and educated generation – and that makes them the most flexible, independent and open to diversity. They are transparent, dynamic and creative. They articulate the need for flexible work programs. Human resources specialists believe that their expectations, when it comes to wage, are unrealistic [14].

### The state of young employees’ mental health

In scientific literature on the mental health of young people, the perspective focusing on young adults as a broad age category dominates, without a clear distinction between those who are economically active and those who are currently not participating in the labor market or have never participated in it. As a result, young employees as a separate study group remain relatively poorly described, even though entering the labor market involves numerous challenges, such as those mentioned in the introduction of this article. The available data on the mental health of young workers is limited, and much of the knowledge in this area does not come from reviewed scientific research, but from surveys and reports prepared by various organizations and institutions involved in labor market analysis. Such data provide valuable and relevant information, but it should be borne in mind that caution is needed when interpreting them, given the lack of detail in the description of the methodology used in the surveys. Nevertheless, due to the lack of data in scientific literature, the results of 4 large surveys will be presented below.

A survey conducted in 2022 by the Mary Christie Institute, located in Lexington, USA, in partnership with several other organizations provides a wealth of important information about the perceived mental health of young employees. The authors presented key findings showing that nearly half of respondents (43%) exhibited symptoms of anxiety and 31% exhibited symptoms of depression. More than half of young workers indicated that they had needed help with their emotions or mental health over the past year. Women reported poorer mental health than men (45% of women said their mental health was good or excellent, compared to 68% of men). Burnout was also a frequently cited problem, with more than half of young workers reporting that they experience it at least once a week. As many as 38% of young professionals believed that their work environment had a negative impact on the well-being and mental health of employees, and almost half of those surveyed believed that it had also negatively affected their own mental health over the past year. Most of them agreed that their workplace should allocate more resources to mental health investments [7].

In 2021, data from the 2015 National Health Interview Survey data was analyzed to examine the prevalence of depression and its impact on healthcare utilization, work absenteeism, and health behaviors among young workers in the United States. Often feeling depressed was reported by 7.8% of respondents, with women being more likely to report this. Depression was most likely to be reported among those working in transportation and material moving occupations (13.0%), construction and extraction (9.9%) and office and administrative support occupations (9.4%). Depression was least common among young workers in production occupations (1.9%) [8].

A 2023 report by the Institute for Employment Studies, based in Brighton, UK, on the mental health of young workers shows that women are more likely to report having a mental health problem or challenge and less likely to inform their employer about it than men. Only 46% of people with mental health problems or challenges decided to disclose this to their employer. Seventeen per cent of them feared the consequences that might result from this. Thirty-seven per cent of respondents indicated that they had struggled with mental health disorders or challenges before starting work, and 7% began to experience them after that [15].

In the 2022 UNISON survey [16], 81% of young employees responded that they had experienced some kind of mental health problem in the past year. Among these people, the most reported problems were depression (88.2%), anxiety (87.1%), but also eating disorders, seasonal affective disorder, post-traumatic stress disorder, obsessive compulsive disorder, and attention deficit hyperactivity disorder. Almost 20% of respondents indicated that their mental health issues were related to work, but the majority (59.3%) indicated that they were only partially related to work; 19.8% responded that their mental health issues were not related to work [16].

### The role of psychosocial risks in young workers’ mental health

Psychosocial occupational risks are elements related to the work performed that can negatively affect an employee's mental and physical health and reduce their overall sense of well-being [17]. When such risks are not adequately eliminated or minimized, their negative effects may lead to a deterioration in the employee's health and well-being and may also impair the functioning of the entire organization in which they are employed.

Research on young employees highlights the impact of several psychosocial work factors on their mental health. The Table 1 summarizes the most frequently reported exposures and outcomes.

**Table 1.** Review of psychosocial risk factors affecting the mental health of young employees: a global evidence summary based on evidence, 2011–2024

Psychosocial risk	Impact on young employees’ mental health
Poor psychosocial job quality (contains low job control) [9,18]	Link to reduced mental health – low levels of control over work were associated with a diagnosis of depression among young employees. Requires further research
Exposure to sexual harassment [9]	It is associated with poorer mental health outcomes; the importance of its co-occurrence with other risk factors is emphasized
Emotional workload and work pressure [19]	Young workers are at a higher risk of mental health problems due to emotional workload compared to older workers; pressure at work affects all age groups, but particularly young workers
Job insecurity (subjective) [20,21]	Subjective job insecurity among young employees is associated with poorer mental health and reduced job satisfaction; the consequences are already apparent at the start of their careers
High demands, effort-reward imbalance, low support [22]	Longitudinal research showed that high demands, effort-reward imbalance and low support (this was only observed among men) were connected to a deterioration in mental health; there is also a correlation between a decline in mental health and the presence of ≥2 risk factors
Precarious work (financial instability, time-related uncertainty, marginal status, employment insecurity) [23]	Precarious forms of employment pose a significant threat to workers’ mental health, particularly among young people who are most likely to experience unstable and temporary working conditions

Although the role of psychosocial work risks in mental health of the whole working population is well described, the associations between these factors and the mental health of young employees remain less clear. While high job demands, job insecurity, low organizational justice, and effort-reward imbalance are reported to be linked to mental health complaints, researchers are uncertain about the causal pathways for young workers [24].

Newer research also suggests that precarious employment, characterized by unstable contracts, unpredictable hours, insufficient income and limited rights, exacerbates feelings of uncertainty and isolation among young people. Such conditions have been associated with heightened stress, anxiety, depression and social withdrawal, disproportionately affecting vulnerable groups such as women, migrants and individuals with limited financial resources [23].

### Mobbing

Mobbing in the workplace represents a critical area of concern within the broader discourse on psychosocial risks, particularly in relation to younger employees entering the labor market. As they often occupy lower hierarchical positions and possess limited professional experience, younger employees may find themselves especially susceptible to hostile behaviors embedded in organizational dynamics. A fundamental element underlying mobbing behavior is the imbalance of power between individuals. Power, in this context, extends beyond physical strength; it encompasses authority, social recognition, and the ability to exert influence over a broader circle of colleagues within the workplace attributes typically associated with seniority and higher effectiveness [25]. Given that seniority and workplace effectiveness often result from greater professional experience and age, younger employees may find themselves disproportionately exposed to this type of pathology in the workplace.

Valuable insights into the scale and nature of this phenomenon among young employees are provided by a study conducted by Jarosz et al. [26] in Poland, which explored the impact of mobbing on individuals entering the labor market, taking into account the size of the organizations in which they are employed. The results revealed no significant differences in the overall prevalence of workplace harassment across organizations of different sizes. However, the most frequently reported manifestations of mobbing included exclusion from decision-making processes and disregard for employees’ ideas and suggestions. These findings underscore the urgent need to recognize mobbing as a pervasive psychosocial hazard that can significantly impair the well-being and professional development of young employees. The early stages of one's career are formative not only in terms of acquiring skills and experience but also in shaping long-term attitudes toward work and organizational culture. Exposure to hostile behaviors during this critical period may lead to lasting psychological distress, diminished job satisfaction, and increased turnover intentions [26]

When it comes to counteracting mobbing, the literature mainly refers to primary prevention measures. Interestingly, these measures can be taken not only in the workplace, but also at the societal level. This primarily involves legal regulations concerning mobbing, which vary from country to country, but also raising public awareness, for example through media campaigns. Various measures can also be taken at the organizational level. There are 4 main areas that are worth focusing on. These are:

– the organization's policy on mobbing,
– implementation of the principles of a ‘safe company’,
– formal regulations concerning provisions in the company's internal regulations,
– procedural and technological facilities that increase safety against exposure to aggression [17].

### The effects of young employees’ mental health problems

Young employees are an important segment of the labor market, as economic dependence on this age group is increasing due to the progressive ageing of the population [27]. The study demonstrated that mental health problems are associated with an increased risk of sickness absence and more frequent absenteeism within this occupational group. Moreover, the findings indicate that mental disorders are linked to reduced productivity, with this effect being particularly pronounced among young employees [28]. In addition, researchers have observed that young workers who show low productivity in the early stages of their careers are more likely to show lower levels of productivity later in their working lives [28].

The phenomenon of unemployment among workers in the age group described is sometimes referred to by researchers as “unemployment among young people” or shorter, “youth unemployment” [29]. This problem has long been the subject of research that highlights its multidimensional and serious consequences in individual and social contexts. Studies have emerged that have shown the economic consequences of youth unemployment for the state [30]. Among the detrimental economic consequences cited are the need to provide unemployment benefits and the underutilization of the country's production and labor force [31], as well as a reduction in the income tax revenue that would be remitted by those who are employed [32]. The absence of an employee can also result for an organization in costs for recruitment, training and adaptation of new employees, replacements, lower productivity and more frequent accidents [33].

### Preventive measures and mental health problems solutions for employers

In response to the prevalence of mental health problems in the young working generation, organizations can implement a number of solutions to prevent and counteract the adverse effects of mental poor health in the younger generations.

Given the heightened vulnerability of younger employees – stemming from their limited authority, experience, and institutional embeddedness – it is imperative that workplace prevention strategies explicitly account for age-related dynamics. Organizations should implement comprehensive anti-mobbing policies, ensure access to supportive structures, and foster inclusive environments where all employees, regardless of age or tenure, feel valued and protected. Prioritizing the prevention of such pathologies is not only an ethical obligation but also a key component of building sustainable and psychologically healthy workplaces [25]. Although it is important to address individual risk factors (Table 2), a holistic approach is recommended.

**Table 2.** Review of psychosocial risks and potential solutions for young employees: a global evidence summary based on evidence, 2010–2025

Psychosocial risk	Possible solutions
Poor psychosocial job quality	The European Youth Forum has identified a number of measures that organizations can implement to effectively support the mental health of young workers. In its report, the organization emphasized the importance of equal rights for workers and of informing young people about their rights. The report also stressed the importance of offering long-term contracts after a probationary period and ensuring regular working hours, as these provide young workers with a sense of stability. Other key factors include wages that reflect real economic conditions and access to social and healthcare programs. The right to be represented by trade unions is also important [23]
	To reduce the negative effects of poor psychosocial workplace conditions on young workers, employers should invest in reliable, accredited training and skills development for their employees. This would allow young workers to take on more challenging and responsible tasks. Skills development would also improve career prospects, reduce the marginalization of young workers, and boost overall job satisfaction. It is important that qualifications acquired lead to opportunities to utilize the acquired skills within the organization [36]
Exposure to sexual harassment	To counteract sexual harassment in an organization, employers must implement a transparent zero-tolerance policy and demonstrate a genuine commitment, going beyond mere compliance with legal standards [37,38]. Training in this area should be implemented and tailored to the specific workplace, including relevant examples of potential behaviors that could constitute sexual harassment. This type of support should be offered regularly, rather than just once [37]. Furthermore, company procedures are essential for preventing sexual harassment, but they must form part of a broader strategy. Effective prevention requires identifying risk factors and reducing the conditions that contribute to sexual harassment. It also requires providing adequate support and response procedures for affected employees [17]. While most countries comply with international regulations, it is crucial that preventive measures are tailored to local workplace conditions [37]
Emotional workload and work pressure	When it comes to counteracting work pressure, there are many options available to organizations. Syahrir and Falaah, in their 2021 article [39], cite research findings that demonstrate the positive impact of interventions based on creating a supportive work environment, for example through the introduction of employee assistance programs and stress management training, but also through the promotion of peer support networks and mentorship programs. The authors emphasize that such practices are particularly valuable in stressful work environments where employees often experience emotional exhaustion. The researchers also cite possible solutions at the level of labor regulations, such as reducing working hours and enforcing recovery periods [39]
Job insecurity (subjective)	Researchers emphasize the important role that communication between employees and employers plays in job security. Sharing relevant information and openness in dialogue can help create a safe environment. Another important factor here is a sense of fairness – there should be a balance between what an employee gives and what they receive [20]. This sense of fairness may stem from various forms of employee participation, which also play an important role in reducing job insecurity by enabling employees to influence the details of decisions made within the organization [40]
High demands, effort-reward imbalance, low support	The results of the studies suggest that it is important for organizations to ensure that employees feel that what they give is adequately rewarded in their work environment. Effort-reward imbalance is associated with significant declines in mental health [41]. In order to support the mental health of their employees, organizations should focus also on fostering employee support – both from the organization, including management, and from other employees [42]. Peer support programs, for example, can be helpful [43]
Precarious work (financial instability, time-related uncertainty, marginal status, employment insecurity)	To protect young workers from the consequences of precarious employment, multi-pronged strategies must be implemented. Organizations should implement measures to mitigate economic risks, ensuring that wages are fair and in line with economic realities [23,44]. It is also important to offer flexible working conditions and guarantee employee benefits. For instance, financial stability could be promoted by establishing employee emergency savings funds [45]. Employees should also receive long-term contracts after their probationary period [23]. Creating a climate conducive to social inclusion and equality in the workplace is also important. Anti-discrimination policies that support employees experiencing economic exclusion and promote employee integration can help build a fair and supportive work environment [46,47]

Many governmental and non-governmental organisations are trying to develop employer-focused recommendations aimed at improving the labor market in line with the needs of young people. One example is Yes Outdoors, a charity operating in London since 2011 that supports vulnerable youth. The organisation has created a practical guide offering targeted interventions [34]. In this guide, Yes Outdoors highlights several key recommendations, including:

– creating a safe and supportive work environment – employers should foster an inclusive and welcoming organizational culture in which young employees feel heard and accepted. Promoting open discussions about mental health plays a vital role in reducing stigma. It is also advisable to implement peer support initiatives and organize group activities that strengthen interpersonal relationships and foster a sense of belonging;
– recognizing symptoms of mental health disorders – it is essential for team leaders and managers to be aware of the typical manifestations of common mental health challenges – such as anxiety, depression, and chronic stress. Attention should be paid to behavioral changes, including sudden mood swings, irritability, absenteeism, or social withdrawal. Encouraging young employees to share their emotional well-being can contribute to building a safe and trustworthy work environment;
– providing early intervention and support – timely responses to signs indicating mental health difficulties are particularly important. Collaboration with mental health support services, educational institutions, and community organizations enables the provision of comprehensive assistance tailored to the individual needs of young employees;
– addressing social and economic challenges – acknowledge the significant influence of housing instability, financial pressure, and family-related difficulties on young people's well-being. Provide support in accessing education, training, and employment opportunities, and advocate for youth-oriented policies that address systemic barriers;
– encouraging healthy lifestyle choices – promote beneficial health behaviors, including regular physical activity, balanced nutrition, and adequate sleep. Encourage young people to engage in creative, sports, and leisure activities, with guidance on digital well-being;
– prioritising self-care for young employees – safeguard personal well-being through peer support and supervision, engage in training related to youth mental health, and establish clear professional boundaries to ensure a sustainable and effective role.

Based on international literature and research findings, researchers have created recommendations for supporting the mental health of young adults in the labor market also for the post-pandemic reality. The authors suggest that it is crucial to proactively use knowledge from 3 main areas:

– nurturing and strengthening protective factors for employee wellbeing, such as managing one's time, working time flexibility or clearly defining expectations and respecting employee boundaries;
– eliminating or reducing psychosocial risks that cause stress, including role ambiguity, limited autonomy or lack of support from superiors;
– implementing solutions tested in the health and social care sector, based on and building a sense of belonging, self-esteem and emotional support in other professional settings.

It is worth considering that, ultimately, this holistic and multifaceted approach, in which employee wellbeing is not entrusted solely to managers or supervisors, but is the responsibility of the whole organization, may prove more effective and efficient [35].

Table 2 outlines the main psychosocial risks affecting young workers and the evidence-based solutions proposed by researchers and organizations. It suggests that, to improve the psychosocial conditions of young workers, a holistic and proactive organizational approach is required. Rather than focusing exclusively on compliance, employers must create environments that promote stability, fairness and inclusion. Key areas for consideration include enhancing job quality by providing meaningful development opportunities, establishing psychological safety and addressing structural inequalities that contribute to insecurity and marginalization. Organizations must foster environments that promote the mental well-being of their employees, ensuring supportive workplace cultures, transparent communication channels and equitable treatment are in place. Such measures are crucial in safeguarding the mental health of young employees and fostering long-term engagement in the workplace.

Although the article focuses on young employees, the good practices proposed can be applied to employees of all ages. Despite the risks listed in the article that particularly affect young people's mental health, preventive measures should be implemented for all members of the organization.

### Synthesis and summary of evidence on psychosocial risks and preventive strategies in young employees’ mental health

The presented narrative overview summarizes the current state of knowledge on various aspects of mental health among young employees, highlighting its multidimensional determinants and key consequences for functioning at the individual and organizational levels. Data from large studies and reports consistently indicate a visible prevalence of mental disorders in this group, while also pointing to a low propensity to disclose mental health issues to employers [7,8,15]. These observations are consistent with international analyses and recommendations that emphasize the importance of organizational measures to prevent psychosocial risks, promote well-being and support employees with existing mental health problems. In this particular context, the global WHO guidelines on mental health at work are of particular importance, as they highlight the necessity for systemic interventions [5].

The present article focuses on a group of young adults as an independent research population and addresses the fact, that there is a gap in the scientific literature, when it comes to analyzing this group separately [24]. From a pragmatic standpoint, the article accurately identifies the optimal prevention strategies as being holistic in nature. These strategies encompass a reduction in psychosocial risk factors, such as high demands, a lack of control, or an imbalance between effort and reward, as well as an enhancement of protective resources. Such resources include social support, organizational justice, and employee participation [17,35]. These observations are consistent with the findings of recent reviews which indicate that interventions focused solely on the individual are inadequate when not accompanied by structural changes in the organizational environment [48].

### Limitations

It is important to take into account the limitations of this article. The narrative nature of the review may involve a risk of bias in the selection of literature. Furthermore, due to the limited number of studies available that meet the criteria set by the authors, some of the empirical data comes from reports and surveys of varying methodological quality, which limits the possibility of drawing conclusions. It is also difficult to separate the effects of age, career stage and intergenerational differences. There are indications in the literature that many of the differences attributed to specific age groups may be better explained by career stage or organizational context than by demographic characteristics [49,50].

Nevertheless, this review confirms that the mental health of young workers is significantly shaped by structural and organizational conditions at work. These findings serve to reinforce the argument for systemic, multi-level preventive measures and underscore the necessity for additional longitudinal research, conducted utilizing a consistent theoretical framework and standardized measures, with a view to facilitating a more profound comprehension of the mechanisms underlying the observed relationships.

## CONCLUSIONS

In light of the above research, it can be concluded that young adults in the workplace have different expectations and needs, and that there is a mental health crisis among young employees. Shaped by rapid technological development, globalization and changing social norms, younger employees from Generations Y and Z have different career preferences that challenge traditional organizational structures.

The reported poor mental health of young professionals is an urgent challenge. Young employees exhibit various types of mental health problems, predominantly those related to depression and anxiety.

Therefore, generational changes require organizational structures to be transformed in a way that is tailored to the needs of young employees. Research shows that psychosocial risks, such as poor psychosocial job quality, exposure to sexual harassment, emotional workload, work pressure, job insecurity, high demands, effort-reward imbalance, low job control, low control and precarious work may be associated with significant mental health complaints and declines. Another important area that is worth focusing on is mobbing, to which young employees are particularly vulnerable due to the fact that they often have lower hierarchical positions and possess limited professional experience.

The organization's perspective on the issue of employee mental health is just as important as the individual's, as it also has economic implications, for example more sickness absences and reduced productivity.

In terms of practical implications, a holistic approach involving the whole organizational structure seems to be the most effective in taking care of mental wellbeing and in counteracting mental health problems in employees. However, despite this, it is also important not to ignore specific risk factors that arise within the organization.

The authors suggest that further research in this area should focus on analyzing the age groups of employees separately, providing preventive measures and solutions that are suitable for the problems that they face.

## AI USE

Translating individual passages from Polish into English, organising bibliographies, and using AI-based search engines for literature review and source exploration.
